# British American Medical and Physical Journal, Montreal, C. W.

**Published:** 1850-07

**Authors:** 


*British American Medical and Physical Journal, Montreal, C. W.* This esteemed contemporary has recently assumed a new and greatly improved typographical appearance. It continues to be edited by Archibald Hall, M. D. Messrs. S. S. & W. Wood, New York, are agents for the United States.

